# Prevalence and associated factors of gender based violence among Baso high school female students, 2020

**DOI:** 10.1186/s12978-021-01302-9

**Published:** 2021-12-14

**Authors:** Alemu Basazin Mingude, Tadesse Mamo Dejene

**Affiliations:** 1 Department of Nursing, Institute of Medicine and Health Sciences, Debre Berhan University, P.O. Box 445, Debre Berhan, Ethiopia; 2 Department of Public Health, Institute of Medicine and Health Sciences, Debre Berhan University, Debre Berhan, Ethiopia

**Keywords:** Gender Based Violence, Determinant, Female students, Debre Berhan, Ethiopia

## Abstract

**Background:**

Gender-based violence (GBV) is a common reproductive health problem, especially in developing countries. It is still the first research priority area in Africa that is identified by World Health Organization. The main aim of this study was to identify the prevalence and determinants of Gender Based Violence among Baso high school female students in Debre Berhan town, Ethiopia.

**Methods:**

An institutional based cross-sectional study was conducted in Debre Berhan, Ethiopia. A total of 350 female students were selected by stratified sampling technique. A self-administered structured questionnaire was used to collect the data. Each independent variable was fitted separately into bivariate logistic analysis, and Variables with p-values less than 0.25 in bivariable model were fitted into multivariate logistic regression analysis to evaluate the degree of association with gender-based violence. The significance level was obtained with 95% CI and p-value < 0.05.

**Result:**

The prevalence of GBV during the lock- down was 36.2% (95% CI 0.3, 0.4), and the lifetime prevalence of GBV was 47.2% (95% CI 0.4, 0.5). The prevalence of life time sexual violence and physical violence were found to be 27.99% (95% CI 0.2, 0.3), and 37.99% (95% CI 0.3, 0.4), respectively. Sexual violence and physical violence during the lockdown were found to be 21.3% and 17.8%, respectively. Respondents educational performance (AOR = 4.5; 95% CI 1.8, 11.3), monthly pocket money received from their parents (AOR = 3; 95% CI 1.6, 5.6), free discussion about reproductive issue (AOR = 2.7; 95% CI 1.4, 5.2), and experience of sexual intercourse (AOR = 13.2; 95% CI 4.8, 36.4) were found to be associated factors of gender based violence.

**Conclusion and recommendation:**

Gender Based Violence is still a significant sexual and reproductive health issue in Ethiopia. Governmental and non-governmental organizations should give due attention to this problem. Moreover, further large-scale studies are needed to estimate the national figure of GBV and to identify route causes.

## Plain Language summary

Gender-based violence (GBV) has been defined as any harmful act that is perpetrated against a person’s will and that is based on socially ascribed (gender) differences between males and females. GBV has a myriad of adverse health effects but is vastly underreported. It is the first research priority in Africa. Every form of violence has devastating effects on the school system such as physical and psychological effects, educational damage and societal breakdown. An institution based cross-sectional study was employed among Baso high school female students. There are four governmental high schools in Debre Berhan town and our study was conducted among Baso high school female students from November 2020–December 2020. The single population proportion formula was used to calculate the required sample size, and the final sample size was 350. First, a stratified random sampling method was employed by considering each grade level as a stratum. Then, a simple random sampling method was used to select the study participants. Study participants were asked about their Sociodemographic characteristics, family history, substance use, sexual experiences, physical violence status, sexual violence status and complications. Out of 350 students, 343 were completed questionnaires, with a response rate of 98%. Lifetime prevalence of GBV was 47.2%.Life time sexual violence and physical violence was found to be 27.99%, and 37.99%, respectively. Respondent’s educational performance, monthly pocket money received from their parents, free discussion about reproductive issue, and experience of sexual intercourse were found to be determinants of gender based violence. In conclusion; Gender Based Violence is still a significant sexual and reproductive health issue in Ethiopia. Significant amount of female students were victim of GBV in our study area.

## Background

Gender-based violence (GBV) has been defined as any harmful act that is perpetrated against a person’s will and that is based on socially ascribed (gender) differences between males and females [1]. It is a pervasive social and public health issue that leads to major physical, psychological and social harm, but it is vastly underreported especially in developing countries [2–4]. The prevalence of gender based violence is high in Sub-Saharan Africa. Ethiopian is the one among sub-Saharan countries which has the highest prevalence of gender based violence (67.7%) [5]. Victims of GBV suffer for stress, anxiety, depression, unsafe abortion, unwanted pregnancy, and sexually transmitted infections [6, 7].

School-Related Gender-Based Violence (SRGBV) is violence or abuse that is based on gendered stereotypes or that target students on the basis of their sex, sexuality, or gender identities [8]. Though violence at school is by no means a new phenomenon, there has been growing social and scientific concern about this issue in recent years [9]. Every form of violence has devastating effects on the school system such as physical and psychological effects, educational damage and societal breakdown [10]. Adolescents who experience school violence more frequently showed a higher risk for feeling of sadness, depression and suicidal ideation [11].

Receiving academic support from male peers, exercising agency in relationship decision-making, having a negative self-concept, belief in stereotypical gender expectations, and engaging in transactional sex and/or substance use were reported risk factors for GBV [12].

Schools are part of society and Reflect traditions and values, at the same time that they play a crucial role in social change [13]. Although schools are recognized as places of learning, personal development and empowerment, they are too often places of discrimination and violence, particularly against female students [14]. A sizeable proportion of the Ethiopian adolescents are enrolled in school. There were 3,466,972 secondary school attendants in Ethiopia in 2019/2020 academic year [15].

Even if, GBV is still the first research priority area in Africa [16]. There were limited studies about gender-based violence in secondary school students in Ethiopia, and no study was found particularly in our study area. Moreover, our study assesses the prevalence of gender-based violence during lockdown due to Covid-19 pandemic. The main aim of this study was to identify the prevalence and associated factor of Gender Based Violence among Baso high school female students in Debre Berhan town, Ethiopia.

## Methods

### Study setting and population

An institution based cross-sectional study was employed among Baso high school female students in Debre Berhan, Ethiopia. Debre Berhan; which is the administrative centre of North Shewa Zone of the Amhara Region, and it is located 130 km far from Addis Ababa, the capital city of Ethiopia. Based on the 2007 national census, this town has a total population of 65,231, of whom 33,563 are women [17]. There are four governmental high schools in Debre Berhan town and our study was conducted among Baso high school female students from November 2020–December 2020. Baso high school was an ideal institution to drown our samples because it contains both urban and rural students.

### Sampling size and determination

**Fig. 1:**
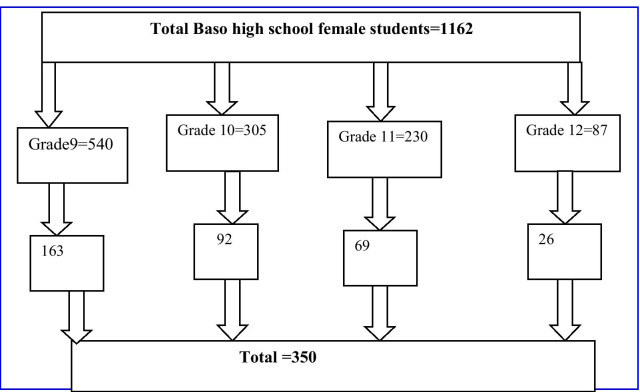
Schematic diagram shows sampling procedure for assessing Gender Based Violence among Basso high school female students, Debre Berhan North Shewa Ethiopia, 2020

Single population proportion formula was used to calculate the required sample size using the lifetime prevalence of sexual violence among high school female students as 68.2% reported in a previous study [18]. Considering 95% CI and 5% margin of error, the final sample size was 350. First, a stratified random sampling method was employed by considering each grade level as a stratum. A list of female students in each grade level was obtained from the registrar office. Then, a simple random sampling method was used to select the study participants (Fig. 1).

### Data collection instrument and procedure

Self-administered structured questionnaire was used to collect the data. The tool was adapted from similar studies conducted in Ethiopia [7, 18]. This assessment tool was developed using English language and then translated to Amharic language and back to the English language to ensure convenient information was retrieved. Questionnaires have different Cronbach's alpha value, i.e. questionnaires related to Sociodemographic characteristics (a = 0.86), questionnaires related to family history (a = 0.76), questionnaires related to substance use (a = 0.72), and questionnaires related to physical and sexual violence status (a = 0.80). Data was collected in calm and separate room. Also, each respondent was left alone when filling the questionnaire to assure their privacy.

### Data processing and analyses

Data were checked for completeness and entered into Epidata version 3.1 and then exported to SPSS version 25 for further data cleaning and analysis. Frequency distributions were obtained to check for data entry error (missing/unrecognized values and codes). Descriptive statistics was done and presented by tables and graphs. The presence of an association between the independent and outcome variable was checked by the Pearson chi-square test. Additionally, each independent variable was fitted separately into bivariate logistic analysis to evaluate for the degree of association with gender-based violence. Variables with p-values less than 0.25 in bivariable model was candidate variable for multivariate logistic regression analysis. In multivariable model, backward stepwise regression was used and variables with p-value < 0.05 in 95% CI were considered independent predictors of GBV.

### Covariates

For each single question participant were responded based on their perception. Followings were questionnaires we used to assess perception. Do you think you are close enough with your parents? (Yes/No), do you receive monthly pocket money from your parents? (Yes/No), how do you perceive your families level of control on you? (1.Tight, 2. Average, 3. Loose/free), and do you have a habit of free discussion about reproductive issues with your partner? (Yes/ No).

## Result

### Sociodemographic characteristics

Out of 350 students, 343 students were completed questionnaires with a response rate of 98%. The other 2% were excluded due to significant incomplete data. The mean age of study participants was 17.03 ± 1.49 years. Majority of the respondents 315 (91.84%) were Orthodox Christian (Table 1).

**Table 1:** Socio demographic characteristics of female Baso high school students in Debre Berhan town, North Shewa Ethiopia, 2020, (N = 343)

Variables	Categories	Frequency (N)	Percentage (%)	Crude OR (95% CI)	P-value
Age	< 18 years	230	67.06	0.7 (0.5, 1.1)	0.143
	≥ 18 years	113	32.94	Ref.	
Religion	Orthodox	315	91.84	1.8 (0.4, 7.3)	0.41
	Protestant	19	5.54	2.2 (0.4, 11.6)	0.34
	Other	9	2.62	Ref.	
Residence	Rural	154	44.9	0.9 (0.6, 1.4)	0.706
	Urban	189	55.1	Ref.	
Living with	Family	268	78.13	1.2 (0.5, 2.6)	0.726
	Husband/boy friend	7	2.04	1.0 (0.2, 5.5)	0.979
	Female friend	12	3.5	1.4 (0.4, 5.4)	0.659
	Relatives	30	8.75	2.4 (0.8, 6.9)	0.118
	Alone	26	7.58	Ref.	
Educational level	Grade 9	159	46.36	0.8 (0.4, 1.9)	0.612
	Grade 10	91	26.53	0.9 (0.4, 2.1)	0.747
	Grade11	68	19.82	0.8 (0.3, 1.9)	0.583
	Grade12	25	7.29	Ref.	
Educational performance	Good and above	126	36.73	Ref.	
	Average	178	51.90	**10.1 (5.7, 17.7)**	**0.001**
	Poor	39	11.37	**6.8 (2.9, 14.2)**	**0.001**
Married/Have boy friend	Yes	29	8.45	0.8 (0.4, 1.8)	0.613
	No	314	91.55	Ref.	
N = 29*****					
Educational status of their husband/boy friend	No formal education	2	6.9	0.6 (0.1, 11.9)	0.718
	Grade 1–8	4	13.79	0.6 (0.1, 5.9)	0.635
	Grade 9–12	12	41.38	0.6 (0.1, 3)	0.511
	Certificate and above	11	37.93	Ref.	
Monthly income status of husband/boyfriend	Yes	16	55.17	1.2 (0.3, 5)	0.837
	No	13	44.83	Ref.	

Bold indicates significant variables

NB: *, total number of respondents (frequency), N*****, total number of respondents who have husband/boy friend

### Family history

Among participants included in this study; 274 (79.89%) of parents were living together. Majority of students 292 (85.13%) had close relation with their parents (Table 2).

**Table 2:** Family history of female Baso high school students in Debre Berhan Town, North Shewa Ethiopia, 2020, (N = 343)

Variables	Category	Frequency (N)*	Percentage (%)	Crude OR (95% CI)	P-value
Family condition	Living together	274	79.89	Ref.	
	Divorced/separated	34	9.91	1.4 (0.7, 2.8)	0.398
	One of them are not alive	27	7.87	1.5 (0.7, 3.4)	0.308
	Both are not alive	8	2.33	2 (0.5, 8.6)	0.344
Educational status of mother	No formal education	122	35.57	1.4 (0.7, 2.7)	0.374
	Grade 1–8	133	38.77	1.0 (0.5, 2)	0.969
	Grade 9–12	44	12.83	0.8 (0.3, 1.8)	0.517
	Certificate and above	44	12.83	Ref.	
Educational status of father	No formal education	112	32.65	1.1 (0.6, 2.1)	0.683
	Grade 1–8	120	34.99	1.7 (0.9, 3)	0.25
	Grade 9–12	44	12.83	0.6 (0.3, 1.4)	0.22
	Certificate and above	67	19.53	Ref.	
Closeness to family	Yes	226	65.9	Ref.	
	No	117	34.1	**13.1 (7.4, 23.4)**	0.001
Pocket money	Yes	236	68.8	**4.2 (2.5, 7.1)**	0.0001
	No	107	31.2	Ref.	
Perceived Family income	Good and above	194	56.56	Ref.	
	Average	118	34.4	0.8 (0.5, 1.3)	0.401
	Poor	31	9.04	1.0 (0.5, 2.1)	0.952
Family control	Tight	223	65	Ref.	
	Average	103	30	0.8 (0.5, 1.2)	0.225
	Loose/free	17	5	**3.5 (1.1, 11.1)**	0.032
Witnessing violence on mother	Yes	34	10	0.7 (0.3, 1.4)	0.289
	No	309	90	Ref.	

Bold indicates significant variables

NB: *, total number of respondents (frequency)

### History of substance abuse

Among our study participants; 86 (25.07%) of them, and 129 (41.98%) of their friends were use Alcohol (Table 3).

**Table 3:** History of substance use among female Baso high school students in Debre Berhan Town, North Shewa Ethiopia, 2020, (N = 343)

Variables	Category	Frequency (N)*	Percentage (%)	Crude OR (95% CI)	P- value
Chew chat (khat)	Yes	10	2.92	0.9 (0.3, 3.1)	0.859
	No	333	97.08	Ref.	
Cigarette smoking	Yes	4	1.17	0.9 (0.1, 6.4)	0.911
	No	339	98.83		
Alcohol drinking	Yes	86	25.07	**4.1 (2.4, 6.9)**	0.0001
	No	257	74.93	Ref.	
Friends chew chat(khat)	Yes	14	4.08	1.6 (0.5, 5)	0.383
	No	329	95.92	Ref.	
Friends’ cigarette smoking	Yes	10	2.92	1.2 (0.3, 5.4)	0.815
	No	333	97.08	Ref.	
Friends drink Alcohol	Yes	114	33.2	**3.1 (2, 5.0)**	0.0001
	No	229	66.8	Ref.	

Bold indicates significant variables

NB: *, total number of respondents (frequency)

### Sexual experience

Among our respondents; 220 (41.14%) had experience of free discussion about reproductive issues with their parents. One hundred-six (30.9%) had sexual experience. From those who had sexual experience; 70 (66.02%) of them were willing at first intercourse (Table 4).

**Table 4:** Sexual experience among female Baso high school students included in this study in Debre Berhan Town, North Shewa Ethiopia, 2020, (N = 343)

Variables	Category	Frequency (N)*	Percentage (%)	Crude OR (95%CI)	P-value
Free discussion about reproductive issues	Yes	220	64.14	Ref.	
	No	123	35.86	**8.7 (5.3, 14.1)**	0.0001
Sexual intercourse	Yes	106	30.9	**33.1 (15.3, 71.9)**	0.0001
	No	237	69.1	Ref.	
N = 106**					
Age at first intercourse	< 18	43	40.57	1.1 (0.52.2)	0.970
	≥ 18	63	59.43	Ref.	
Willingness at intercourse	Yes	70	66.02	Ref.	
	No	36	33.98	3 (0.2, 37.7)	0.395
Number of sexual partners	One	66	62.26	0.7 (0.1, 4.2)	0.668
	Two	21	19.81	0.7 (0.1, 5.3)	0.759
	Three	14	13.21	0.7 (0.1, 5.3)	0.702
	Four	5	4.72	Ref.	

Bold indicates significant variables

NB: *, total number of respondents (frequency), N**, total number of respondents who have experience of sexual intercourse

### Magnitude of gender based violence

The lifetime prevalence of GBV, and GBV during the lock down was 47.2% (95% CI 0.42, 0.53) and 36.2% (95% CI 0.31, 0.41) respectively. Life time sexual violence and physical violence were found to be 27.99% (95% CI 0.23, 0.33), 37.99% (95% CI 0.33, 0.43), respectively. Sexual violence and physical violence during the lockdown were found to be 21.28% and 17.78%, respectively (Table 5).

**Table 5:** *Sexual violence among female Baso high school students included in this study* in Debre Berhan Town, North Shewa Ethiopia, 2020, (N = 343)

Variables	Category	Frequency (N)*	Percentage (%)
Lifetime sexual violence	Yes	96	27.99
	No	247	72.1
Sexual violence before joining school	Yes	45	13.12
	No	298	86.88
Sexual violence since joining school	Yes	65	18.95
	No	278	81.05
Sexual violence in academic year	Yes	28	8.16
	No	315	91.84
Sexual violence during lock down	Yes	73	21.28
	No	270	78.72
N = 96***			
Perpetrator of sexual violence	Boyfriend/Husband	13	13.54
	Family member	18	18.75
	Teacher	24	25.00
	Student	41	42.71
Place of sexual violence happened	Home	60	62.5
	Hotel	31	32.29
	Another place	5	5.21
Share incident to family	Yes	18	18.75
	No	78	81.25
Reporting to legal body	Yes	9	9.37
	No	87	90.63
N = 87****			
Reason for not reported	Didn’t know what to do	13	14.94
	Fear of perpetrator	19	21.84
	Feeling of shame	55	63.22

NB: *, total number of respondents (frequency); N***, total number of respondents who have experience of sexual violence; N****, victims of sexual violence who did not report to legal body/parents

### Physical violence

**Fig. 2:**
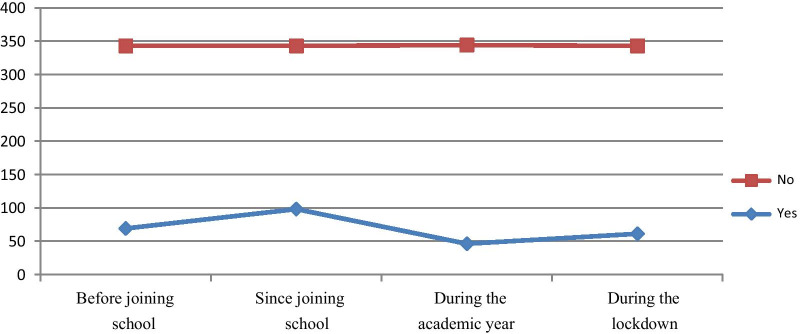
prevalence of physical violence among Basso high school students, 2020

Of all respondents, 130 (37.9%) reported physical violence once in their lifetime. Among respondents 69 (20.12%) reported that violence happened before joining the school, 98 (28.57%) after joining the school and 46 (13.14%) sustained during this academic year (Fig. 2).

### Sexual violence and perpetrators

The lifetime prevalence of sexual violence among female students was found to be 27.99%, while sexual violence before joining the school, after joining the school and in the current academic year was reported as 45 (13.12%), 65 (18.95%) and 28 (8.16%) respectively. According to our study, the offenders of physical violence were family members, students, teachers and husbands/boyfriends (Table 5).

### Consequences of sexual violence

**Fig. 3:**
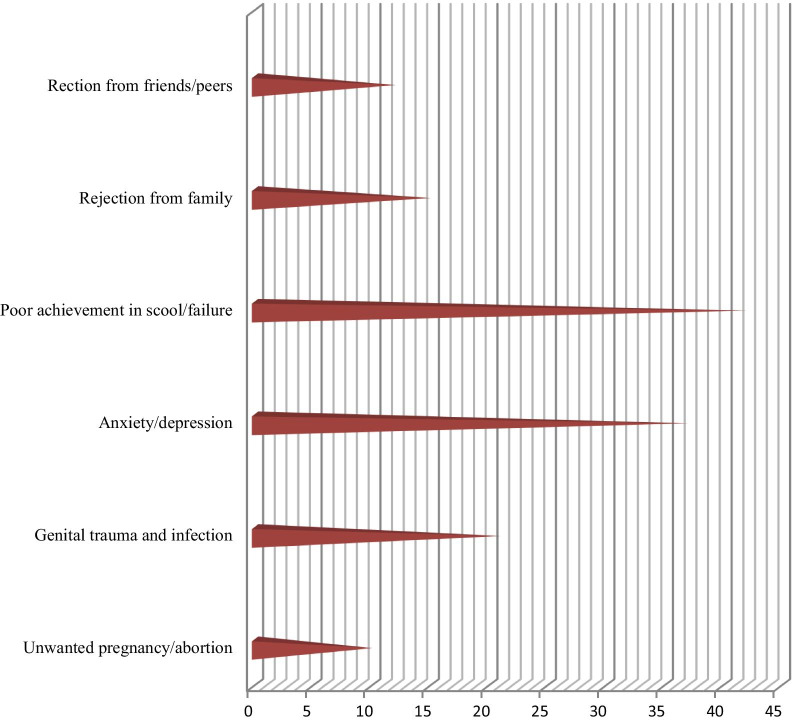
Effect of sexual violence on the victims among Basso high school female students in Debre Berhan, Ethiopia, 2020

There were lots of reported physical, psychological and other complications associated with sexual violence. Complications like: rejection from family, rejection from friends/peers, poor academic achievement/ failure to continue the school, unwanted pregnancy, abortion, infection and trauma to genital area (Fig. 3).

### Factors associated with gender-based violence

Initially, bivariate logistic regression analysis was conducted to identify variables found to be associated with GBV. Based on this, respondents who have poor educational performance (COR = 6.7; 95% CI 2.9, 14.2), respondents who were not close enough to their family (COR = 13.1; 95% CI 7.4, 23.1), respondents who receive pocket money (COR = 4.2; 95% CI 2.5, 7.1), Loose family control (COR = 3.5; 95% CI 1.1, 11.1), respondents who drink alcohol (COR = 4.1; 95% CI 2.4, 6.9), respondents who have friends who drink alcohol (COR = 3.1; 95% CI 2, 5), respondents who have not experience of free discussion about reproductive issues (COR = 8.7; 95% CI 5.3, 14.1),and respondents who have previous sexual experience(COR = 33.1; 95% CI 15.3, 71.9) have more association with gender based violence.

Next, multivariate logistic regression analysis was employed to control confounders and to identify independent factors associated with GBV. The multivariate analysis results show that; respondents who have poor educational performance, respondents who receive monthly pocket money from their parents, respondents who have not experience of free discussion about reproductive issues, and respondents who have previous experience of sexual were found to be associated factors of gender-based violence (Table 6).

**Table 6:** Factors associated with gender-based violence among Basso high school female students, 2020

Variables	Category	Frequency	COR (95% CI)	AOR (95% CI)	P-value
Educational performance	Good and above	126	Ref.	Ref.	
	Average	178	10.1 (5.7, 17.7)	1.6 (0.5, 5.6)	0.235
	Poor	39	6.7 (2.9, 14.2)	**4.5 (1.8, 11.3)**	0.001
Closeness to family	Yes	226	Ref.	Ref.	
	No	117	13.1 (7.4, 23.1)	2.7 (0.7, 11)	0.163
Pocket money	Yes	236	4.2 (2.5, 7.1)	**3 (1.6, 5.6)**	0.001
	No	107	Ref.	Ref.	
Family control	Tight	223	Ref.	Ref.	
	Average	103	0.8 (0.5, 1.2)	0.8 (0.2, 3.3)	0.785
	Loose/free	17	3.5 (1.1, 11.1)	0.6 (0.1, 2.9)	0.564
Alcohol drinking	Yes	86	4.1 (2.4, 6.9)	1.2 (0.5, 2.8)	0.768
	No	257	Ref.	Ref.	
Friends who drink alcohol	Yes	114	3.1 (2.0, 5.0)	1.7 (0.8, 4.6)	0.671
	No	229	Ref.	Ref.	
Free discussion about reproductive issues	Yes	220	Ref.	Ref.	
	No	123	8.7 (5.3, 14.1)	**2.7 (1.4, 5.2)**	0.003
Sexual intercourse	Yes	106	33.1 (15.3, 71.9)	**13.2 (4.8, 36.4)**	0.00001
	No	237	Ref.	Ref.	

Bold indicates significant variables

Ref., Reference; COR, Crude Odd Ratio; AOR, Adjusted Odd Ratio; CI, Confidence Interval

Respondents who have poor educational performance were more than 4 times more likely to gender based violence than respondent who have good educational performance(AOR = 4.5; 95% CI 1.8,11.3). The second variable which had association with GBV was monthly pocket money, respondents who receive monthly pocket money from their parents were 3 times more likely for gender based violence than their counterpart (AOR = 3; 95% CI 1.6, 5.6). The other variable which had association with GBV was free discussion about reproductive issues, respondents who have not experience of free discussion about reproductive issues were almost 3 times more likely for gender based violence than their counterpart (AOR = 2.7; 95% CI 1.4,5.2). Previous experience of sexual intercourse also had association with GBV, respondents who have previous experience of sexual intercourse were more than 13 times more likely for gender based violence than their counterpart (AOR = 13.2; 95% CI 4.8, 36.4).

## Discussion

Gender Based Violence is a practice of intentional harm to people usually considering gender and using traditional gender inequalities, and it is the worldwide problem it is against human right of peoples and has many consequences on health related complications as well as life-threatening outcomes it has so many forms like physical, sexual and psychological violence particularly against women.

### Magnitude of gender based violence

In our study, the lifetime prevalence of gender-based violence was 47.2% and the gender-based violence particularly during the period of lockdown was 36.2%. The prevalence of sexual violence was 27.9% and physical violence was 37.9%. At the time of lock-down, the prevalence of sexual violence and physical violence was found to be 21.28% and 17.78% respectively. This finding is consistent with a study conducted in Debre Markos city particularly the lifetime gender-based violence were reported to be 47.0% which exactly a similar result but in terms of sexual violence our study result showed a higher prevalence compared with a study done in Debre Markos city, which was 23.3% and regarding physical violence our result was slightly lower that the results of Debre Markos city which was 39.5% [19]. The similarities in lifetime gender-based violence might be due to the nature of similarities of culture, and social living status of the participants because both of the study areas are from Amhara region and mainly Amhara ethnicity the differences might be due to sample size.

Our study finding was lower than a study conducted in the southern part of Ethiopia (wolaita Sodo and Aleta wondo), and a study conducted in Maputo. A cross sectional study which was published recently indicated that the prevalence of life time gender based-violence (GBV), sexual and physical violence were 63.2%, 37.2%, and 56.3%, respectively in Wolaita Sodo town and another study in Aleta wondo reported that lifetime prevalence of gender based violence were 68.2% as well as sexual and physical violence were 26.3% and 56.14% respectively the study conducted in Maputo reported that the lifetime prevalence of gender based violence among school adolescent girls were 55.7% the difference for this might be due to the difference in socio cultural and norms among the study populations besides the difference also might be due to study settings [7, 18, 20].

Regarding gender based violence during the lockdown; there were studies on domestic violence which strengthen our findings. The prevalence of sexual violence during the lockdown were 33.8% in Ethiopia, 22% in Uganda, [21, 22]. Study in Tunisia, Africa, and the Arab world indicates the increment of gender based violence from 4.4% to 14.8% during the lock down [23]. There is also study conducted in Ethiopia on intimate partner violence which supports our study. The prevalence of sexual violence on this study was 5.3% and age less than 30 (AOR = 23.1; 95% CI 5.6,94.4) were one of the associated factor [24].

GBV was not a new scenario, but increased in severity and incidence during the lock down [25]. This is due to the fact that; the pandemic and controlling measures (like school closure) had contributed to the furtherance of gender based violence and child abuse. Let’s for ample, we see the case of two regions in Ethiopia, Amhara and Addis Ababa which was reported by Association for Human Rights in Ethiopia. In Amhara region there were 203 rape cases (of which 54 are child victims), 208 Physical assault (of which 192 are women and 8 are child victims, 30 homicides (of which 25 are women and 5 are child victims) for the period between April to June 2020. Parents in rural areas of the region also forced to their minor children to marry believing that schools will never reopen or stay closed for a long time. This contributed to the escalation of child marriage in rural areas. Out of 3613 child marriage reports to the Regional General Attorney, 845 were proved to be child marriage and cancelled by police intervention. In addition, during the lockdown women were forced to remain home with violent partners, which in turn led to the rise in domestic violence cases. Apart from intimate partner violence, women were highly exposed to rape. In case of Addis Ababa; there were similar cases related to lockdown. Data from a few hospitals in Addis Ababa showed that, between mid-March and mid-May 2020, more than 100 girls were raped, some by close family members. In the same period, 50 women were exposed to domestic violence [26].

### Associated factors of gender based-violence

From the findings of this research educational performance, pocket money, the free discussion about reproductive health issues, and experience of sexual intercourse showed significant associations with the outcome variable which is gender-based violence.

Poor educational performance among high school students were associated with gender-based violence in our study those who have poor educational status has 4.5 times more at risk of having gender based violence or experienced gender-based violence than their counterparts these finding shows similarities with the study conducted at Bahirdar city high school female students according to the study those students who had poor educational performance were three times the odds of gender-based violence than those who have good school performance [27]. Another study conducted in Menkorer High school, North west Ethiopia indicated that respondents with current educational status of good and above were less likely to be a victim of gender based violence by nine Percent when compared with students who have poor educational status [6]. Another study conducted in Mombasa, Kenya showed that literacy decreased gender based violence’s [28].

Regarding the student’s economic status or their pocket money which they get from their families are related to their gender-based violence situation; in our study we found that those students who have pocket money were more likely to expose for gender-based violence than their counterparts. This finding is contradicted from other research findings like a study conducted in, Dilla, south Ethiopia and a study conducted in Gondar, northern Ethiopia. Their finding indicates that having pocket money reduces the risk of gender based violence [29, 30]. This may be due to the positive association between the amount of money young people received and higher rates of drug use [31, 32] that results increase in risk of gender based violence.

Those students who do not have the free discussion about reproductive health issues had 2.7 times odds of gender-based violence than their counterparts this finding is supported by the study conducted in other areas of the country a study conducted at Bahirdar city in Amhara region reported that, lack of experience of discussion about sexual and reproductive health issues with their family and or friends has increased almost four times the odds of GBV as compared to their counterparts [27]. Another study conducted at Gondar showed that significant association between the outcome variable gender based violence and the independent variable of free discussion about reproductive health issues [30]. This may be due to the fact that discussion of sexual and reproductive health issues between parents and adolescents is one of the best ways that enable adolescents to delay sexual activity and create awareness to prevent violence.

Another significant variable that shows association with the outcome variable in our study is sexual intercourse so, according to our finding, those students who have a history of sexual intercourse has 13.2 times the odds of getting gender-based violence than their counterparts. This finding was supported by study conducted in Dilla which revealed that those students who have sexual experience were 5.7 times more likely getting gender based violence than their counterparts [29]. There were also other studies reported the association of sexual activity with gender based violence among high school students [7, 33].

## Strength and limitation of the study

We have assessed one of the major issues; gender based violence specifically during the lockdown and provides relevant information’s. However, this study has some limitations. The first limitation was the nature of the study design, i.e. cross-sectional study; it will not tell us time association between the factors and the outcome variables. The second limitation was the study conducted on single institution which makes difficult its generalizability.

## Conclusion and recommendation

The prevalence of overall gender-based violence, sexual, physical, and emotional violence was high. This study also found that respondent’s educational performance, monthly pocket money received from their parents, free discussion about reproductive issue, and experience of sexual intercourse were strongly associated with gender-based violence. A comprehensive educational institution-based prevention strategy and effective interventions should be developed to mitigate gender-based violence. Government policymakers, non-governmental organizations, program designers and other stakeholders should be developed effective intervention and prevention strategies to reduce gender-based violence in educational institutions.

## Data Availability

The datasets used and/or analysed during the current study are available from the corresponding author on reasonable request.

## Abbreviations

AOR: Adjusted odd ratio
CI: Confidence interval
COR: Crude odd ratio
GBV: Gender based violence
IRB: Institutional Review Board
WHO: World Health Organization

## References

[1] WHO Guidelines Approved by the Guidelines Review Committee. Inter-agency field manual on reproductive health in humanitarian settings: 2010 Revision for field review. Geneva: Inter-agency Working Group on Reproductive Health in Crises Copyright © 2010 Inter-agency Working Group on Reproductive Health in Crises; 2010.

[2] Nature S. Understanding gender-based violence: an essential textbook for nurses, healthcare professionals and social workers. 2021.

[3] Palermo TBJ, Peterman A (2014). Tip of the iceberg: reporting and gender-based violence in developing countries. Am J Epidemiol.

[4] Garcia-Moreno C, Jansen H, Ellsberg M, Heise L, Watts C, Naved R (2006). Prevalence of intimate partner violence: findings from the WHO Multi-country Study on Women's Health and Domestic Violence. Lancet.

[5] Beyene AS, Chojenta C, Roba HS, Melka AS, Loxton D (2019). Gender-based violence among female youths in educational institutions of Sub-Saharan Africa: a systematic review and meta-analysis. Syst Rev.

[6] Mullu G, Gizachew A, Amare D, Alebel A, Wagnew F, Tiruneh C (2015). Prevalence of gender based violence and associated factors among female students of Menkorer high school in Debre Markos town, Northwest Ethiopia. Science.

[7] Tantu T, Wolka S, Gunta M, Teshome M, Mohammed H (2020). Duko BJBph Prevalence and determinants of gender-based violence among high school female students in Wolaita Sodo, Ethiopia: an institutionally based cross-sectional study. BMC Public Health.

[8] Tkach K, Ahn J, Gonzalez NV, Xu Z, Zhang Y. The effects of school-related gender-based violence on academic performance: Evidence from Botswana, Ghana, and South Africa; 2016.

[9] Pulido Valero R, Martín Seoane G, Lucas MB (2011). Risk profiles and peer violence in the context of school and leisure time. Span J Psychol.

[10] Lee E (2019). Effects of school violence on depression and suicidal ideation in multicultural adolescents. Iran J Public Health.

[11] Silva BRVS, Silva AO, Passos MH, Soares FC, Valença PA, Menezes VA (2018). Negative self-perceived health associated with school violence in adolescents. Ciencia Saude Coletiva..

[12] Kaufman MR, Williams AM, Grilo G, Marea CX, Fentaye FW, Gebretsadik LA (2019). "We are responsible for the violence, and prevention is up to us": a qualitative study of perceived risk factors for gender-based violence among Ethiopian university students. BMC Womens Health.

[13] USAID. Unsafe schools: a literature review of school-related gender-based violence in developing countries; 2003.

[14] Smith PKJViS. Violence in schools: an overview. 2004; pp. 17–30.

[15] Federal Ministry of Education EaID. Education Statistics Annual Abstract; 2020.

[16] Ouedraogo L, Nkurunziza T, Muriithi A, John TK, Asmani C, Elamin H (2020). The WHO African Region: research priorities on sexual and reproductive health and rights. Adv Reprod..

[17] Ethiopia Nsao. Population and housing census 2007 Report, Amhara, Part I: population size and characteristics; 2007.

[18] Dogiso A, Shegaze M, Alagaw A, Wassihun B (2019). Prevalence and associated factors of gender-based violence among high school female students in Aleta Wondo Town, south east Ethiopia. Ethiopian J Reprod Health..

[19] Ashebir W, Ayichew A. Associated factors of sexual and gender based violence among female high school students in Debre Markos Town, North West Ethiopia: an institutionally based cross-sectional study; 2021.

[20] Maguele MS, Tlou B, Taylor M, Khuzwayo NJ (2020). Risk factors associated with high prevalence of intimate partner violence amongst school-going young women (aged 15–24years) in Maputo. Mozambique..

[21] Nabukeera M (2020). Prevention and response to gender-based violence (GBV) during novel Covid-19 lock-down in Uganda. J Adult Protection..

[22] Tesfaw LM, Kassie AB, Flatie BT (2021). Sexual violence and other complications of corona virus in Amhara Metropolitan Cities, Ethiopia. Risk Manag Healthcare Policy..

[23] Sediri S, Zgueb Y, Ouanes S, Ouali U, Bourgou S, Jomli R (2020). Women's mental health: acute impact of COVID-19 pandemic on domestic violence. Arch Womens Ment Health.

[24] Gebrewahd GT, Gebremeskel GG, Tadesse DB (2020). Intimate partner violence against reproductive age women during COVID-19 pandemic in northern Ethiopia 2020: a community-based cross-sectional study. Reprod Health.

[25] Fawole OI, Okedare OO, Reed E (2021). Home was not a safe haven: women's experiences of intimate partner violence during the COVID-19 lockdown in Nigeria. BMC Womens Health.

[26] Belete SS. The impact of COVID-19 on human rights in ethiopia. association for human rights in Ethiopia; 2021.

[27] Belay HG, Liyeh TM, Tassew HA, Ayalew AB, Goshu YA, Mihretie GN (2021). Magnitude of gender-based violence and its associated factors among female night students in Bahir Dar City, Amhara Region, Ethiopia. Int J Reprod Med.

[28] Bhattacharjee P, Ma H, Musyoki H, Cheuk E, Isac S, Njiraini M (2020). Prevalence and patterns of gender-based violence across adolescent girls and young women in Mombasa, Kenya. BMC Womens Health.

[29] Desalegn T (2014). Assessment of the prevalence and associated factors of sexual violence among high school female students in Dilla town, Gedeo zone SNNPR.

[30] Birkie M, Zenebe Y, Biset G, Gebresellassie M, Mihret S (2020). Risk factors for gender-based violence among female students of Gonder Teacher’Training College, Gonder, Northwest Ethiopia: a cross-sectional study. Open Public Health J..

[31] Gebremariam TB, Mruts KB, Neway TK (2018). Substance use and associated factors among Debre Berhan University students, Central Ethiopia. Subst Abuse Treat Prev Policy.

[32] McCrystal P, Percy A, Higgins K (2007). The cost of drug use in adolescence: young people, money and substance abuse. Drugs Educ Prev Policy..

[33] Heslop J, Parkes J, Johnson Ross F, Alito F, Turner E. The Code of Conduct on prevention of school-related gender-based violence: a study of policy enactment in Ethiopia; 2019.

